# Unraveling the Complications: Stent Migration and Duodenal Fistula in a Metastatic Desmoplastic Round Cell Tumor

**DOI:** 10.14309/crj.0000000000001997

**Published:** 2026-02-04

**Authors:** Michael Cymbal, Arjun Chatterjee, Renan Prado, Leandro Sierra, Stephen A. Firkins, Roma Patel, Akash Khurana, Roberto Simons-Linares

**Affiliations:** 1 Internal Medicine Residency Program, Cleveland Clinic, Cleveland, OH; 2 Department of Gastroenterology, Digestive Diseases and Surgery Institute, Cleveland Clinic, Cleveland, OH

**Keywords:** migrated biliary stent, over the scope clip, OTS clip, duodenal fistula, renal perforation

## CASE REPORT

**Figure 1.**
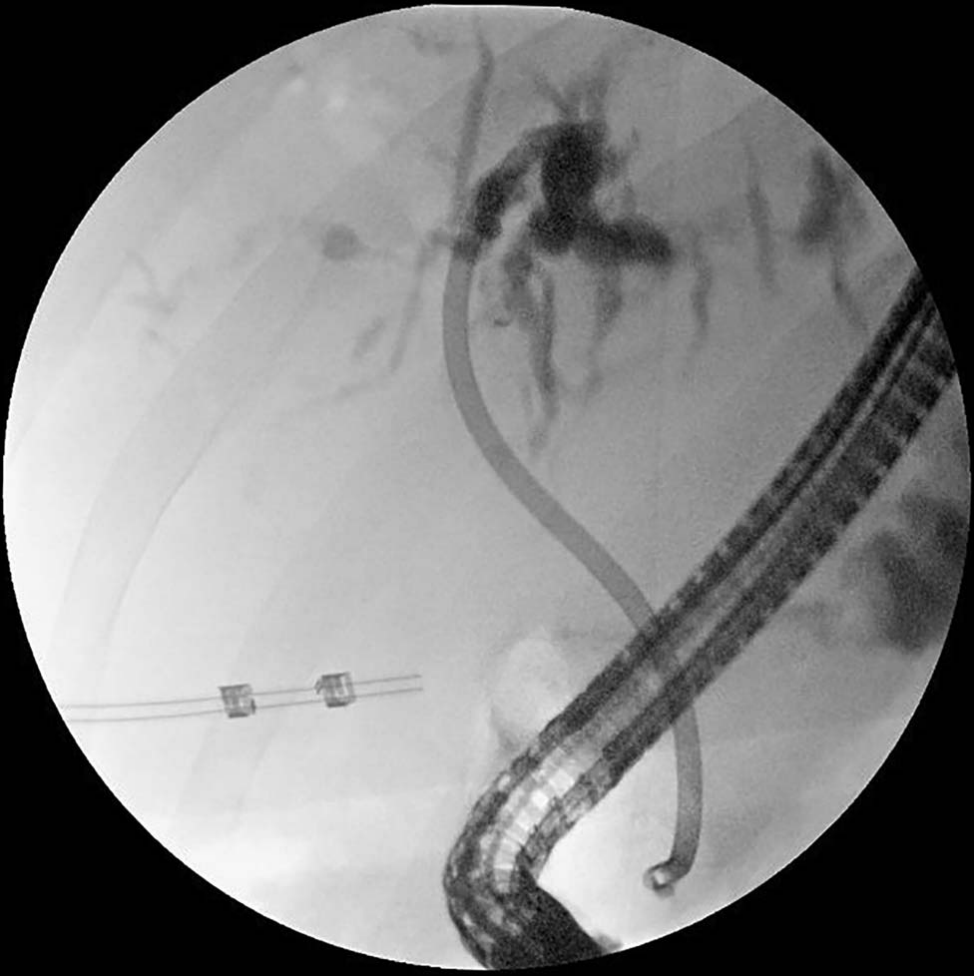
Endoscopic retrograde cholangiopancreatography revealed choledocholithiasis and a biliary stricture at the hepatic duct bifurcation, which was dilated and stented.

**Figure 2.**
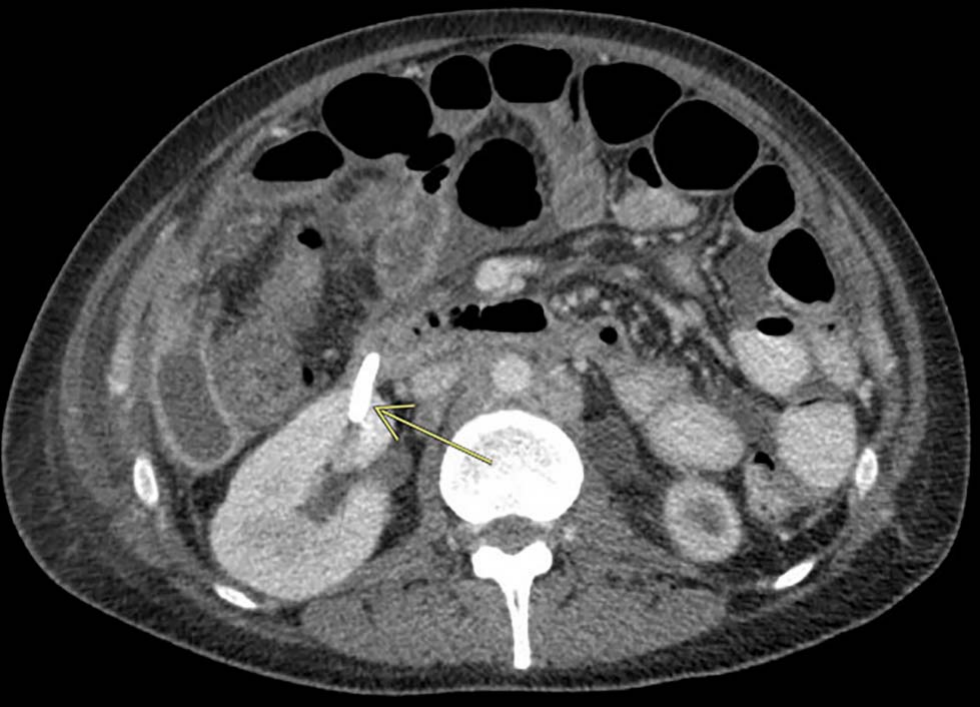
Computed tomography of the abdomen revealed that the plastic biliary stent had penetrated the posterior duodenal wall and right renal cortex, creating a 5-mm fistula.

Biliary stent migration is a recognized complication of endoscopic retrograde cholangiopancreatography.^1–5^ Although plastic biliary stent migration occurs in approximately 5%–10% of cases, intestinal perforation is rare, with duodenal involvement reported in <1%.^1^ We report a 30-year-old man with metastatic desmoplastic round cell tumor who required a 10Fr × 12-cm plastic biliary stent for a malignant stricture (Figure 1). Two months later, he presented with right upper quadrant pain and sepsis; imaging demonstrated gallbladder perforation, which was managed conservatively with percutaneous drainage and antibiotics. Four months after initial placement, the stent migrated, penetrating the posterior duodenal wall and right renal cortex, resulting in a duodenorenal fistula (Figure 2).

**Figure 3.**
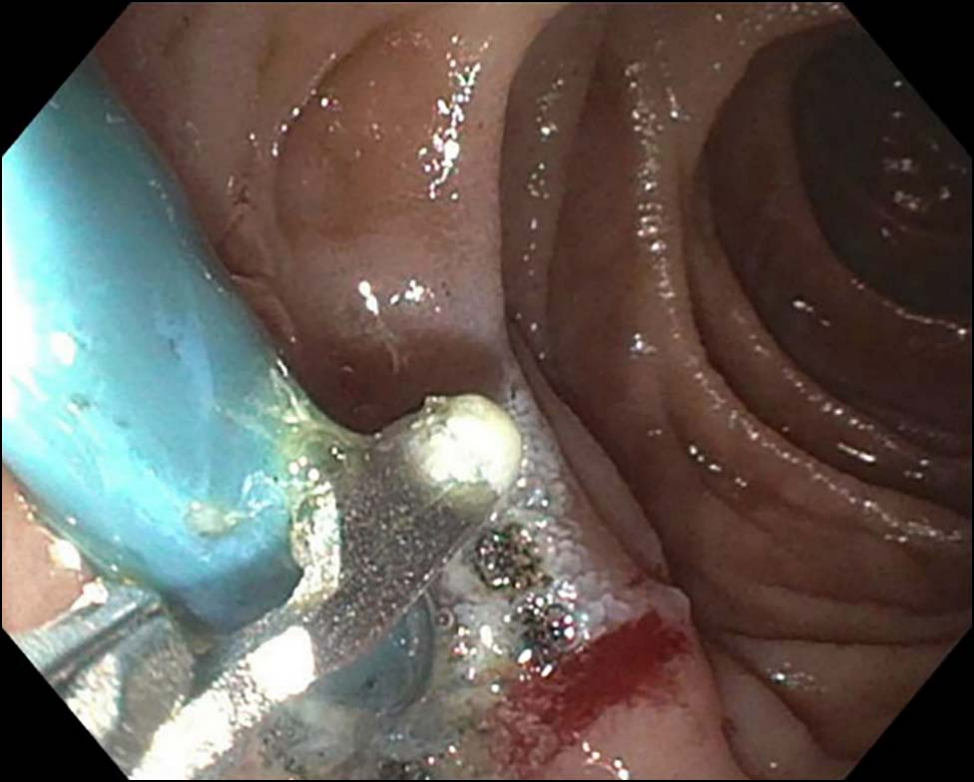
Upper endoscopy revealing plastic biliary stent had penetrated the posterior duodenal wall.

**Figure 4.**
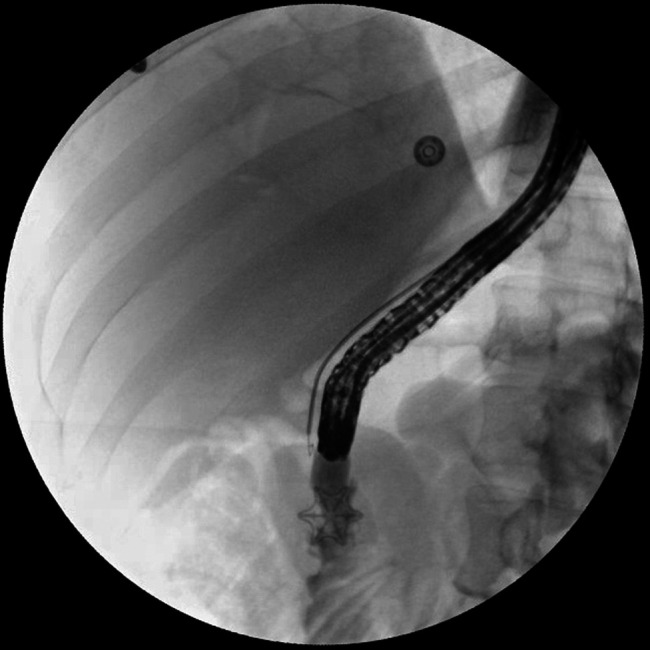
Enterogram confirming no extravasation after placement of the over the scope clip for fistula closure.

**Figure 5.**
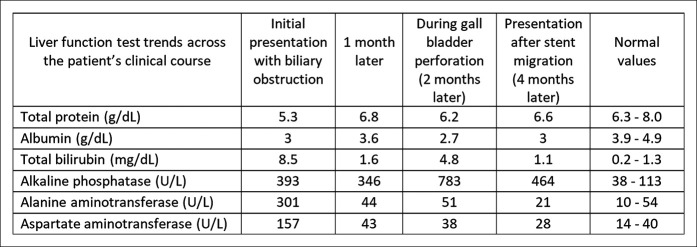
Liver function test trends across the patient's clinical course.

Subsequent imaging and endoscopic retrograde cholangiopancreatography confirmed the fistula. Endoscopic management was successfully performed with removal of the migrated stent, closure of the duodenal fistula using suturing and an over-the-scope clip, and placement of an uncovered metal stent in the common bile duct to restore biliary drainage (Figures 3–5; Video 1). The patient was discharged 2 days later without complications.

This case highlights the rare but serious complication of duodenal perforation from biliary stent migration and demonstrates that complex perforations involving adjacent organs can be effectively managed endoscopically.^1–5^

## DISCLOSURES

Author contributions: M. Cymbal; A. Chatterjee: drafting the article. R. Prado; L. Sierra; SA Firkins; R. Patel; A. Khurana: critical revision of the manuscript. R. Simons-Linares: final approval of manuscript and is the article guarantor.

Financial disclosure: None to report.

Informed consent was obtained for this case report.
